# Cyclic Peptide Stabilized Lead Halide Perovskite Nanoparticles

**DOI:** 10.1038/s41598-019-49643-7

**Published:** 2019-09-10

**Authors:** Anna Jancik Prochazkova, Yolanda Salinas, Cigdem Yumusak, Oliver Brüggemann, Martin Weiter, Niyazi Serdar Sariciftci, Jozef Krajcovic, Alexander Kovalenko

**Affiliations:** 1 Linz Institute for Organic Solar Cells (LIOS), Physical Chemistry, Johannes Kepler University Linz, Altenbergerstraße 69, 4040 Linz, Austria; 2 Faculty of Chemistry, Materials Research Centre, Brno University of Technology, Purkyňova 118, 612 00 Brno, Czech Republic; 3 Institute of Polymer Chemistry (ICP), Johannes Kepler University Linz, Altenbergerstraße 69, 4040 Linz, Austria

**Keywords:** Nanoparticles, Organic-inorganic nanostructures

## Abstract

Combining the unique properties of peptides as versatile tools for nano- and biotechnology with lead halide perovskite nanoparticles can bring exceptional opportunities for the development of optoelectronics, photonics, and bioelectronics. As a first step towards this challenge sub 10 nm methylammonium lead bromide perovskite colloidal nanoparticles have been synthetizes using commercial cyclic peptide Cyclo(RGDFK), containing 5 amino acids, as a surface stabilizer. Perovskite nanoparticles passivated with Cyclo(RGDFK) possess charge transfer from the perovskite core to the peptide shell, resulting in lower photoluminescence quantum yields, which however opens a path for the application where charge transfer is favorable.

## Introduction

The incorporation of electronic functionality into biological molecules opens a pathway for bioelectronics nanomaterials. Among others, proteins and peptides are the most versatile biological molecules due to their extensive chemical, conformational and functional diversity^1^. To apply the “bottom-up” approach in which self-assembly occurs at a molecular/atomic level plays a crucial role to develop biomolecular nanostructures for emerging bioelectronic technologies^2–5^.

Inspired by nature, solid-binding peptides have been developed and became ubiquitous molecular building blocks in nanotechnology^6–8^. These peptides show selectivity and bind with high affinity to the surfaces of a wide range of solid materials including metals, metal oxides and compounds, magnetic materials, semiconductors, polymers, and minerals. They can direct the assembly and functionalization of materials, and have the ability to mediate the synthesis and construction of nanostructures^9^.

The extensive research is devoted on the different applications of self-assembling peptides for the next generation of biosensors, as well as functional electrochemical and optoelectronic devices^10,11^. These materials are even considered as alternative semiconductor materials because of their bio-friendly nature, morphologically advantageous, and cost effective easy preparation, modification and doping^12^. Moreover, it was found that especially self-assembled nanostructures constituted of short peptides show optical characteristic of quantum dots^13^ and exhibit quantum confinement effect^14^ with tunability of the luminescence from visible to near-infrared spectral range^15^.

On the other hand, due to the noncovalent interactions, including hydrogen-bonding, π−π stacking, van der Waals interactions, and electrostatic interactions, self-assembled peptides exhibit highly dynamic bonding nature and this makes them combine with other class of materials such as lead halide perovskites. Lead halide perovskite are candidates in electronics and optoelectronic devices due to their tunable band gap^16^, large absorption coefficient^17^, long diffusion length for both types of carriers^18^ and ease of processability^19^. Due to the aforesaid, lead halide perovskites have found use in various devices such as: photovoltaics^20–23^, light emitting diodes^23,24^, photodetectors^25^, etc^26^. In addition, high crystallization ability from precursor solution allows to obtain a large variety of nanostructures^27^, where the surface chemistry governs the formation of the nanoparticles^28^.

In the present work, we have attempted to combine lead halide perovskite nanoparticles and peptides by stabilizing perovskite surface with cyclic pentapeptide containing 5 amino acids: Arginine, Glycine, Aspartic acid, Phenylalanine and Lysine (Cyclo(RGDFK)). This is the first step towards combining unique properties of solid-binding peptides and perovskite nanoparticles. Cyclic peptide was chosen regarding its higher stability with a respect to the linear counterparts^29^.

## Materials and Methods

**Figure 1:**
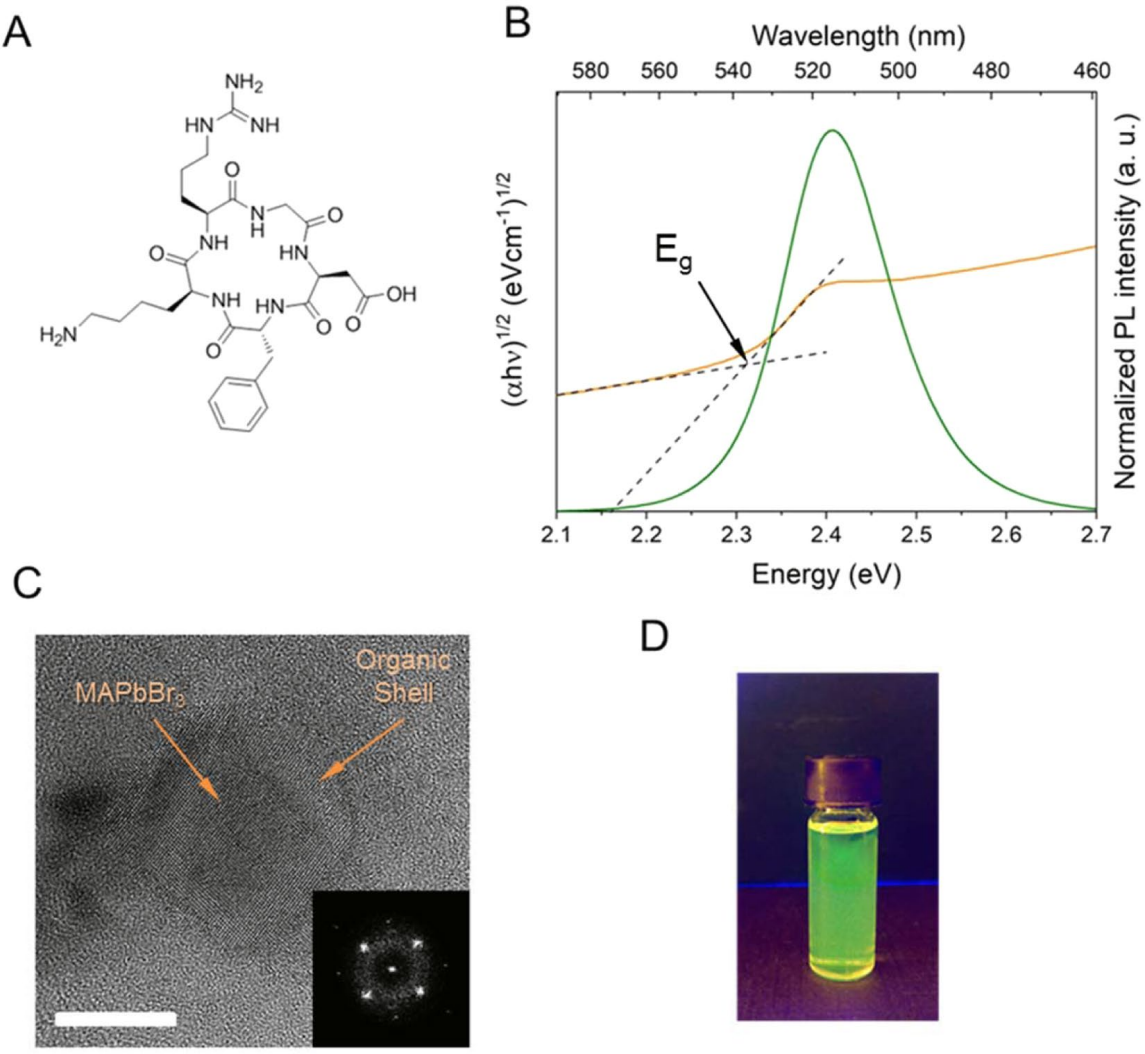
Cyclic peptide Cyclo(RGDFK) (**A**) used in the perovskite nanoparticle formation; Optical characteristics of the colloidal solutions: Photoluminescent spectra and Tauc absorption plots (**B)**; TEM images of corresponding PNPs with FFT pattern inset (scale bar 5 nm) (**C**); Photograph of the lead halide nanoparticles’ colloidal suspensions in chloroform under UV illumination (**D**).

Lead (II) bromide (PbBr_2_ 99.999%) and Methylammonium bromide (MABr, 98%) were purchased from Sigma Aldrich. were purchased from Alfa Aesar. Hexanoic acid (98%) was purchased from TCI. Cyclo(Arg-Gly-Asp-D-Phe-Lys) (Fig. 1A) (≥97% HPLC) was purchased from Sigma-Aldrich. Solvents - dimethyl sulfoxide (DMSO) and chloroform was of reagent grade (≥99.8%), purchased from VWR. All chemicals were used as received without any further purification.

The synthesis of colloidal solution was performed by ligand assisted precipitation method^30^. Perovskite precursors: lead (II) bromide (PbBr_2_), methylammonium bromide (MABr), hexanoic acid (HeA) and Cyclo(RGDFK) in molar ratios 1:1.1:9.5:0.6 dissolved in DMSO was precipitated in chloroform. For the precipitation of PNPs the vial with chloroform was placed in the ice bath (at 0–3 °C) with a stirrer. Then 20 μm of the precursor solution were added drop-wise to the vial with 10 ml of chloroform. It has to be noted, that in case if no Cyclo(RGDFK) was added in the precursor solution orange sediment consisting of cubic microcrystals of MAPbBr3 was formed, as reported previously^31^.

As an additional cleansing step the colloidal solution was centrifuged (5000 rpm/10 min), then the supernatant was discharged and the resulting solid was re-dispersed in chloroform. It is worth to be noted, that ultraviolet-visible (UV-VIS) and photo luminescent (PL) optical spectra of crude and washed colloidal dispersions of nanoparticles didn’t show any significant changes, which is an evidence that Cyclo(RGDFK) can stabilize dynamic perovskite surface.

Ultraviolet-Visible (UV-Vis) and fluorescence spectroscopy were used to characterize the optical properties of the PNP. UV-Vis spectroscopy was carried out with a Lambda 1050 UV/Vis/NIR spectrometer (PerkinElmer). Photoluminescent (PL) spectra were taken on a fluorimeter from Photon Technology International. UV-Vis and PL spectra of colloidal PNP suspensions were measured in a 3 mL quartz cuvette at λ_EXC_ = 405 nm.

TEM images were obtained with a Jeol JEM-2200 FS microscope using Holey Carbon film 300 mesh Copper grids. All samples grids were treated for 5 min in a Jeol EC-52000IC Ion cleaner before the TEM measurement. FT-IR spectra were recorded on a Perkin-Elmer Spectrum 100.

For the geometry optimization was applied Gaussian 09^32^ software package with B3LYP functional extended by 6-311 + G* with split-valence polarized triple-ζ basis set polarization and diffuse functions on heavy atoms for C, N, H and O atoms and Los Alamos National Laboratory 2 Double-ζ (LANL2DZ) for Pb and Br atoms. The SCRF procedure was applied to take into account effect of the solvent (DMSO). Force constants and the resulting vibrational frequencies were computed in order to simulate IR spectra of the complexes.

Photoluminescence quantum yields (PLQY) measurements were performed via “direct excitation” method also used by S. Wang *et al*.^33^. The PLQY was determined as ratio between the number of emitted photons and the number of absorbed ones. Measurement was performed in the integrating sphere in a fluorometer (Photon Technology International) with λ_EXC_ = 405 nm in a 3 mL quartz cuvette.

The absolute PLQY (η) were calculated according to the equation

$$\eta =\frac{{E}_{{\rm{B}}}}{{S}_{{\rm{A}}}-{S}_{{\rm{B}}}}$$

where *E*_B_ is the area under the curve in the emission part of the spectra and *S*_A_ and *S*_B_ are excitation areas of the sample and pure solvent, respectively.

## Results and Discussion

**Figure 2:**
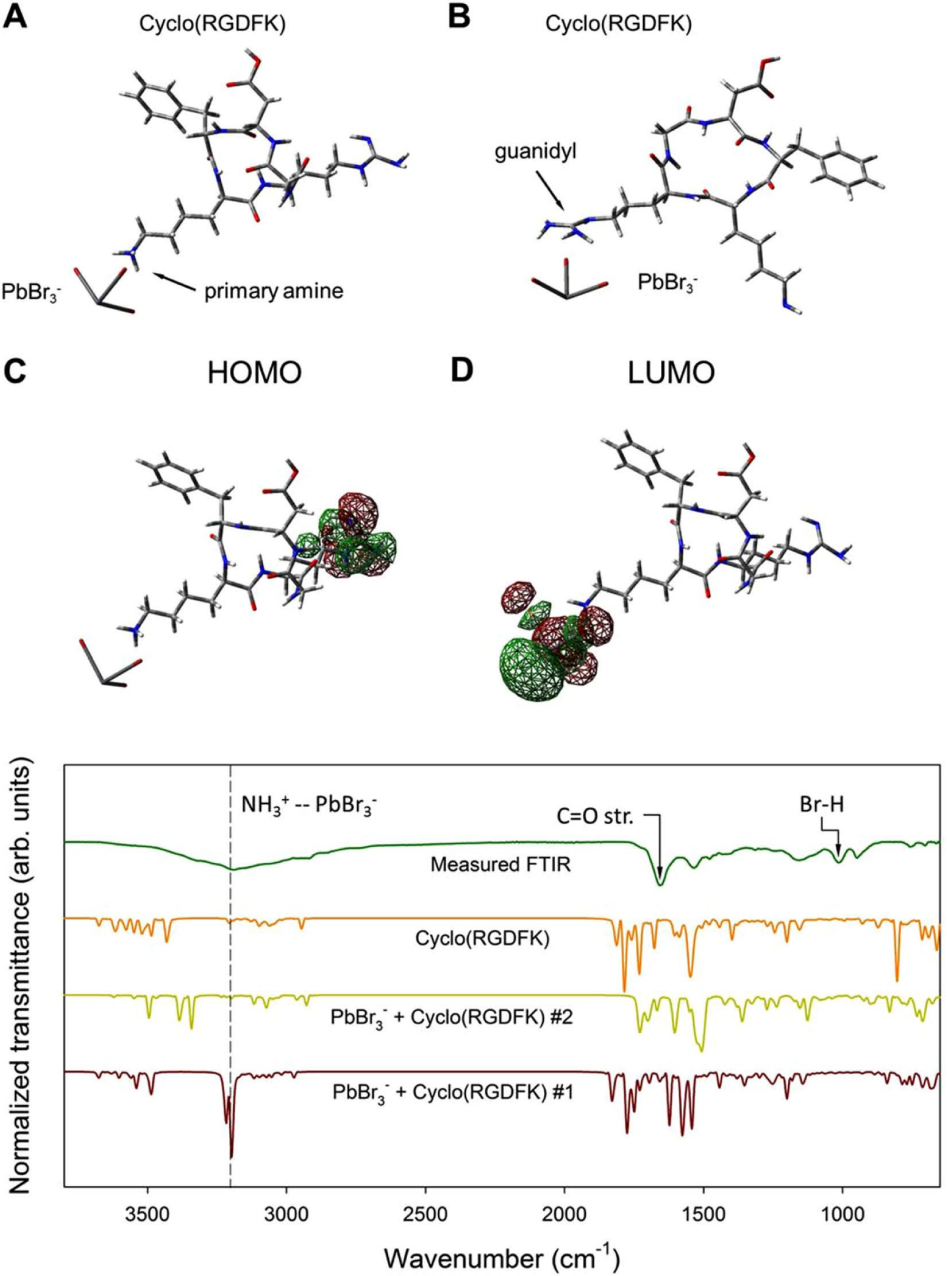
B3LYP/6-311 + G* Optimized geometries of the PbBr_3_^−^/Cyclo(RGDFK) precursor complexes, (**A**) – where the peptide is coordinated to the PbBr_3_^−^ with the primary amine group and (**B**) where the coordination bond is formed between PbBr_3_^−^ and guanidyl group; Isosurfaces of electron density distribution at HOMO (**C**) and LUMO (**D**) electronic orbitals in the complex (**A**); FTIR spectra show typical absorption peaks that are attributed to the presence of peptide ligands on the surface of nanoparticles.

As a result a green emitting (515 nm) colloidal dispersion was obtained. It possessed a nearly negligible Stockes shift (absorption maximum at 514 nm). Optical band gap was estimated as 2.3 eV from the Tauc plot (Fig. 2B) considering direct band gap in lead halide perovskites^33^. Photoluminescence quantum yields (PLQY) measured by an integration sphere^33^ was about 20%, which is significantly lower than the most of previously reported values for perovskite nanoparticles^34^, which can be associated with reabsorption^35^ or electron transfer as discussed below.

For the evaluation of the nanoparticles size, transmission electron microscopy (TEM) was applied. For the measurements colloidal solution of perovskite nanoparticles was drop casted onto the copper grid and dried in ambient conditions. TEM images (Fig. 1C) show a collection of particles with an average diameter d = 6 ± 2 nm. Taking into the account bright dots of the Fourier transform (FFT) pattern and highly defined crystalline planes on TEM images it can be stated that the samples are highly crystalline. Diffuse rings of the FFT pattern are coming from the carbon film of the TEM grid.

To get a deeper insight about the coordination complexes in the precursor solution, which is a key locus for the formation of nanoparticles, we carried out quantum chemical calculations. Interestingly, low degree of complexity modeling can be used to predict preferential generation of species under the effect of the solvent. Long or bulky molecules containing amino groups are intended to passivated nanoparticles’^30^ surface by interruption of the lattice crystallization in the antisolvent medium. In the present case, Cyclo(RGDFK) assumingly binds to the perovskite surface, thus forming nanoparticles.

Thus coordination complexes of PbBr_3_^−^/Cyclo(RGDFK) were modeled. Given the presence of both amine and guanidyl groups in Cyclo(RGDFK), two possibilities of coordination complexes were considered (Fig. 2A,B): on the one hand Cyclo(RGDFK) can be bounded to the perovskite surface with the amino group and on the other hand guanidyl group can form the coordination bond with lead bromide. Taking into account identical number of electrons in both models, the figure of merit was Gibbs free energy for both of the complexes, and therefore it had been observed, that the energy difference between the complex A and the complex B is 0.39 eV in a favor of complex A, which indicates, that the complex A is more probable candidate for the nanoparticle formation. Moreover, from the optimized geometry, in can be observed, that specific shape of complex B, can sterically hinder further crystallization of the nanoparticles.

In correspondence with previously reported work^36^ we applied FTIR spectroscopy to evaluate the complex formations. From the measured FTIR spectra it can be observed, that characteristic broad peak at ~3200 cm^−1^ highly corresponds to the simulated vibrations of the ammonium group NH_3_^+^ in the vicinity of PbBr_3_- (Fig. 2E, spectrum #1) in case of complex A. Notably, this peak is absent in the simulated spectra of both complex B (Fig. 2E, spectrum #2) and bare Cyclo(RGDFK). In accordance with the aforesaid, it can be stated that the complex where cyclic peptide coordinated to the PbBr_3_^−^ with the amino group is preferential for the nanoparticle formation.

Interestingly, concerning the electron density distribution in the complex A, it has been observed, that in the ground state the electron at the highest occupied molecular orbital (HOMO) is localized at the guanidyl group. However, the electron density at the lowest unoccupied molecular orbital (LUMO) is displaced to the PbBr_3_^−^ (Fig. 2C,D). Considering, that the perovskite nanoparticles are formed in a manner, that the guanidyl groups are not coordinated to the perovskite structure and directed *ectad*, considering dominant hole mobility in the methylammonium lead bromide perovskite^37^ it can be assumed, that during the photo excitation the charge carrier transfer can take place between the nanoparticle and surface peptide ligands, which in turn can be the reason of the reduced PLQY in comparison with previously reported results^34,38–40^.

## Conclusion

To sum up, in the present communication, we demonstrate as first an efficient way of stabilizing the surface of methylammonium lead bromide perovskite nanoparticles with cyclic peptide Cyclo(RGDFK). Green emitting sub-10 nm nanoparticles, as it is confirmed by TEM, are obtained in a form of colloidal dispersion. Interestingly, the nanoparticles can be centrifuged, washed and re-dispersed without any significant changes in spectral properties. By the comparison of measured FTIR with vibrational analysis of modeled low complexity systems we have shown, that the nanoparticles are most likely formed with the guanidyl group of the Cyclo(RGDFK) is directed outwards. Lower luminescent yields can be the result of the charge carrier transfer between the nanoparticle core and peptide shell. Indeed, the deeper study of the simulated complexes shows that the electron tends to be displaced from the peptide towards the perovskite core. In the present case lower PLQY one the hand can be disadvantageous for the application in light emitting devices, however on the other hand using the peptide properties to be attached to the selected surfaces such nanoparticles can be applied in the devices, where charge transport is favorable (i.e. sensors). In general, the above-described approach can open a door towards numerous possibilities of combining the unique properties of solid-binding peptides as molecular building blocks in nanotechnology and perovskites as promising materials for applications in electronics.
